# Evidence for a non-stochastic two-field hypothesis for persistent skin cancer risk

**DOI:** 10.1038/s41598-020-75864-2

**Published:** 2020-11-05

**Authors:** Raymond L. Konger, Lu Ren, Ravi P. Sahu, Ethel Derr-Yellin, Young L. Kim

**Affiliations:** 1 Department of Pathology and Laboratory Medicine, Indiana University School of Medicine, 975 West Walnut Street, IB424F, Indianapolis, IN 46202 USA; 2 Department of Dermatology, Indiana University School of Medicine, Indianapolis, IN USA; 3 Department of Pathology, Richard L. Roudebush Veterans Administration Hospital, Indianapolis, IN USA; 4 Department of Pharmacology and Toxicology, Boonshoft School of Medicine, Wright State University, Dayton, OH USA; 5 Weldon School of Biomedical Engineering, Purdue University, West Lafayette, IN USA

**Keywords:** Cancer microenvironment, Cancer models, Skin cancer, Squamous cell carcinoma

## Abstract

With recurring carcinogen exposures, individual tumors develop in a field of genetic mutations through a stepwise process of clonal expansion and evolution. Once established, this “cancer field” persists in the absence of continued carcinogen exposures, resulting in a sustained risk for cancer development. Using a bioimaging approach, we previously demonstrated that a dermal premalignant field characterized by inflammatory angiogenesis persists following the cessation of ultraviolet light exposures and accurately predicts future overlying epidermal tumor formation. Following ultraviolet light treatments, others have observed that patches of p53 immunopositive cells persist stochastically throughout the epidermal stem cell population. However, these studies were done by random biopsies, introducing sampling bias. We now show that, rather than being randomly distributed, p53^+^ epidermal cells are enriched only in areas overlying this multi-focal dermal field. Moreover, we also show that the dermal field is characterized by a senescent phenotype. We propose that persistence of the overlying epithelial cancerization field in the absence of exogenous carcinogens or promoters requires a two-field composite consisting of a dermal senescent field driving the persistence of the overlying epidermal cancer field. These observations challenge current models that suggest that persistence of cancer risk in the absence of continued carcinogen exposures is simply a function of stochastically arranged, long-lived but dormant epithelial clonal stem cells mutants. The model proposed here could provide new insights into how cancer risk persists following cessation of carcinogenic exposures.

## Introduction

Exposure to ultraviolet (UV) light from the sun is the primary cause of human skin cancer, which frequently occurs as multiple lesions^1^. The relatively high frequency of multiple malignant lesions within a carcinogen-treated epithelial field was originally termed ‘field cancerization’ by Slaughter et al.^2^. Field cancerization studies have largely focused on genetic and epigenetic changes to the epithelial cells undergoing neoplastic transformation^3,4^. Tumor development within the field has been theorized to be dependent on the positive selection of clones that outcompete normal or benign clonal variants, leading to clonal expansion, thereby increasing the chances for the acquisition of additional necessary mutations for full tumorigenic conversion^3^. Evidence for this epithelial mutation field was recently documented in geriatric sun-damaged skin^4^. Using ultradeep sequencing, known driver mutations for cutaneous squamous cell carcinoma (cSCC) were found in 18 to 32% of otherwise normal cells^4^. This included mutations of tumor protein p53 (*TP53*), a driver mutation that is frequently found in sun-exposed epidermis, and is enriched in premalignant and malignant skin lesions^4^. Mutations in the tumor suppressor protein p53 generally result in loss of p53 proteolytic degradation and nuclear accumulation^5^. Thus, clonal “patches” of cells harboring p53 mutations can be readily visualized as p53^+^ cells by immunohistochemistry.

In mice treated with ongoing UV treatments, p53^+^ patch formation is shown to increase exponentially, eventually merging to form large fields^6,7^. Since humans are often only periodically exposed to UV rays or minimize their risk through sunscreen or sunblock use, this model of ongoing carcinogen treatment fails to mimic common human behavior. To better examine how the epithelial field changes following UV avoidance strategies, mouse models have examined p53 clonal patch resolution following cessation of repetitive UVB exposures^6,7^. Following UVB cessation, terminal differentiation and tissue renewal are thought to result in a rapid decline of approximately 60–90% of the p53 clonal patches over the initial few weeks^6,7^. The remaining patches are then found to persist. The persistence of these patches are thought to be dependent on p53 mutations within randomly distributed epidermal stem cells that are present throughout the UV treated field^6^. Since early p53^+^ clonal populations lack cell autonomous proliferative potential, it has been proposed that these mutant stem cells remain dormant until subsequent carcinogen or tumor promoter exposures occur to reignite the clonal expansion and evolution process^7^. This persistence of the epithelial field is also consistent with observations that skin cancer risk persists in individuals long after ceasing UV exposures^8^.

Assessing the early premalignant field in the past has been difficult due to the inability to directly visualize the field. In the absence of field visualization, studies are limited to random biopsies that in turn contribute to the idea that the epidermal mutation field is randomly distributed to the stem cell niche. Thus, a key novel attribute of our approach is the use of a non-invasive imaging methodology that visualizes and maps the premalignant dermal cancer field^9^. Using our imaging approach, 96% of tumors occurred in areas of pre-existing hyperemia that represent a small fraction of the carcinogen-treated area^9^. Importantly, we demonstrated that once developed, the dermal hyperemic field persists and expands in the absence of any additional UV treatments or exogenous promoters. Moreover, these hyperemic foci formed well before malignant lesions developed that could drive their own tumor microenvironment. While this study focused on the dermal field, the occurrence of tumors almost exclusively in areas overlying the dermal field suggests that persistence of the epithelial mutation field may not be dependent on a stochastic arrangement of clonal stem cell mutations within the epidermal field, but may be dependent on changes with the dermis. Moreover, it was not clear how these dermal foci persist and expand in mice long after UV treatments have ceased.

Studies have clearly shown that stromal changes induced by exogenous tumor promoters, including UV, can promote overlying epidermal clonal field expansion and tumorigenesis^7,10^. Yet, it is unclear why cancers continue to develop after removal of exogenous promoting signals. While established tumors have the ability to alter the stromal microenvironment to provide a supportive microenvironment^11–13^, it remains to be determined how early small populations of clonal pre-malignant cells that lack the ability to promote their own growth continue to undergo clonal evolution in the absence of continued promoter or carcinogen exposures. One possibility is suggested by the ability of UV treatments to promote skin aging, resulting in an accumulation of senescent cells within the dermis. Senescent fibroblasts are known to produce pro-inflammatory and mitogenic mediators that could stimulate clonal proliferation and expansion^14,15^. Given that senescent cells are long-lived^16^, are resistant to apoptosis^16^, and produce angiogenic, mitogenic and inflammatory mediators^14,15^, a senescent stroma could serve as a driver for the persistence of the dermal field and the continued clonal expansion and evolution of the overlying epithelial field.

The idea that senescent stromal cells can stimulate tumor growth is not new. Co-injection studies show that senescent fibroblasts can promote the growth, invasiveness and metastasis of tumor cells via paracrine signaling^14,15^. However, these studies also utilize very large numbers of tumor cells that differ significantly from the early premalignant field, in which clonal populations of premalignant cells can be limited to 10’s or 100’s of cells. Moreover, as noted above, while invasive tumors have the capacity to alter the stromal microenvironment to a more conducive environment for tumor growth and invasion, this capacity is not thought to be present in early small benign premalignant cell clone populations.

The best evidence that the stroma can promote tumorigenesis in the absence of exogenous carcinogens or tumor promoters is seen in mice with mesenchymal loss of recombination signal binding protein for immunoglobulin kappa J region (RBP-Jκ)^17^. In this study, mesenchymal RBP-Jκ(–/–) mice exhibited multi-focal areas of inflammation and epidermal hyperplasia (assessed by 5-aminolevulinic acid (5-ALA) uptake), and developed spontaneous squamous tumors^17^. While the authors show that loss of RBP-Jκ in fibroblasts could result in a senescent phenotype, the study did not demonstrate a spatial correlation between senescent stromal features and the overlying 5-ALA uptake.

Given our ability to map out the dermal field early in the premalignant field, the goal of this current study was to determine whether the persistent dermal field observed following cessation of UV treatments was associated with p53^+^ patch retention and whether dermal senescence was a characteristic feature of the hyperemic foci. Following the cessation of UV treatments, both epidermal p53^+^ patches and epidermal hyperplasia were seen to persist *only* in areas bounded by the hyperemic dermal field. Moreover, a senescent dermal cell phenotype was enriched *only* in hyperemic areas at high risk for subsequent tumor formation and p53^+^ patch retention.

## Results

### Bioimaging

**Figure 1:**
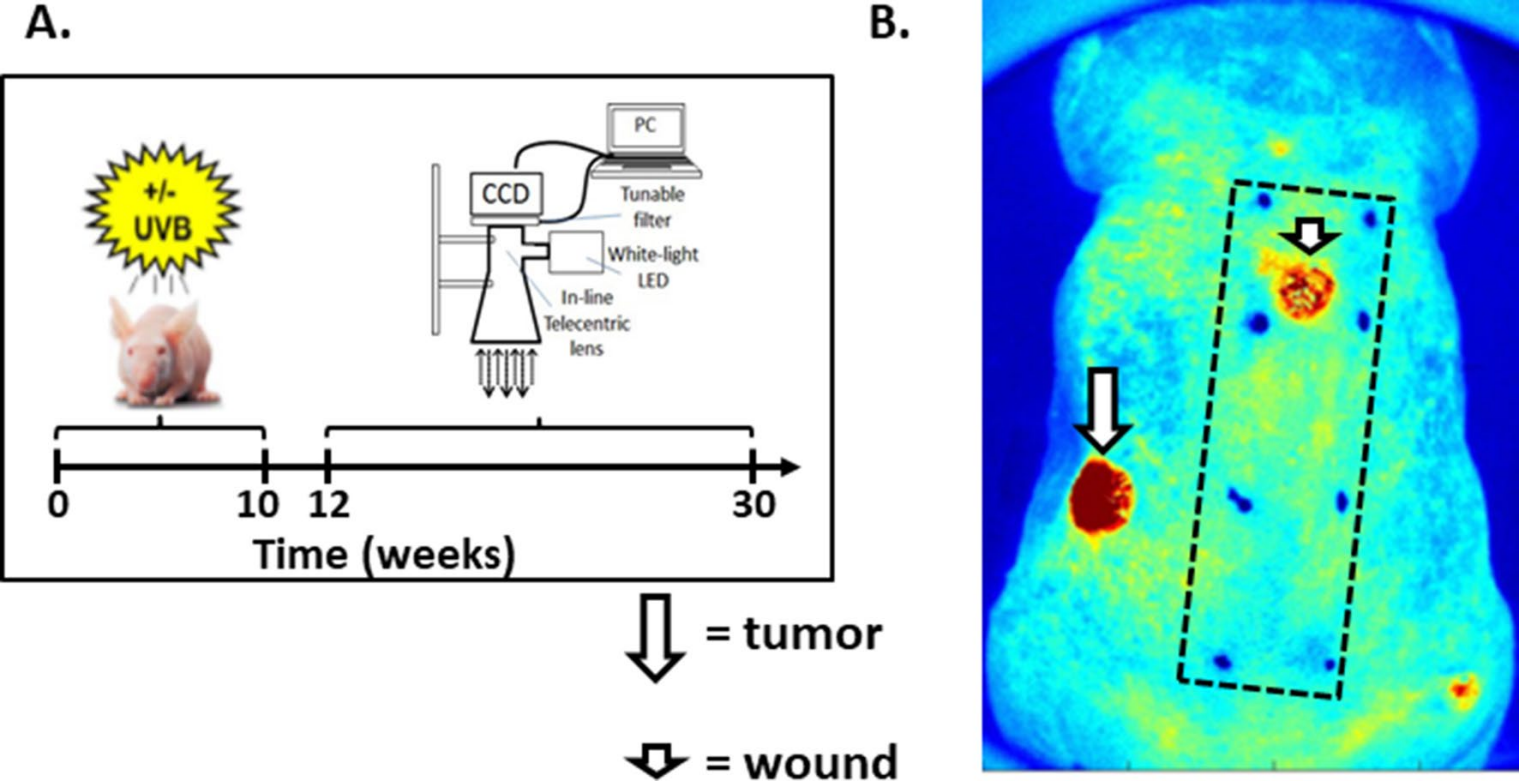
An improved bioimaging device to quantify and map superficial hemoglobin (Hgb) content. (**A**) *Depiction of the experimental timeline.* SKH-1 hairless albino mice were treated thrice weekly with 2240 J/m^2^ of UVB for 10 weeks. The UVB treatments were then stopped and the mice were imaged to generate Hgb content maps using a 2nd generation telecentric lens imaging system. Telecentric lenses provide constant perspective across the field of view and constant image magnification. More importantly, they act as back-directional (angle) gating (or filtering). (**B**)*. Field of view using the 2nd generation imaging device with telecentric lens.* Using a telecentric imaging approach, a Hgb content map was imaged in the same mouse as that used for the conventional lens system in (**B**). The image size now included most of the dorsal surface of the mouse. The long arrow shows the intense increase in Hgb content within a papilloma that is outside the field of view using the conventional imaging method. The short arrow depicts Hgb content in a wound. The boxed area represents the field of view using our previous 1st generation imaging system.

Figure 1A depicts our experimental model for UVB-induced carcinogenesis. SKH-1 mice were treated with UVB for 10 weeks. The UV treatments were then discontinued. We then performed image analysis of the mice for dermal hyperemia at 2 and 20 weeks after discontinuing UV treatments. In our previous study, hemoglobin (Hgb) content within the superficial dermis was assessed using a fixed camera, conventional lens, and fixed light paths to allow for back-directional angle gating^9^. The limitations of this design were that the imaging device was immobile and had a relatively limited field of view (approximately 1.5 cm square). This required digital stitching software to obtain images covering an approximately a 1.5 cm × 4.5 cm section of the UV treated mouse dorsal epidermis (see hatched rectangle in Fig. 1B). For these current studies, we utilized a new mobile imaging device that takes advantage of the optical properties of a telecentric lens to provide constant perspective, image magnification, and back-directional gating^18^. The improved field of view is demonstrated by the image shown in Fig. 1B.

### UV-induced persistent dermal hyperemic foci correlate with persistent overlying epidermal hyperplasia

Following the cessation of a carcinogenic dose of UV, we previously observed that subsequent tumor formation occurs almost exclusively within preformed areas of persistent hyperemia^9^. This suggests that the epithelial cancer field persists only in areas overlying dermal hyperemia. Since epidermal hyperplasia is a consistent marker of tumor promoter treatment and is thought to be necessary for clonal expansion of premalignant epithelial cells, we examined whether epidermal hyperplasia persists in the epidermis overlying hyperemic dermal foci.

**Figure 2:**
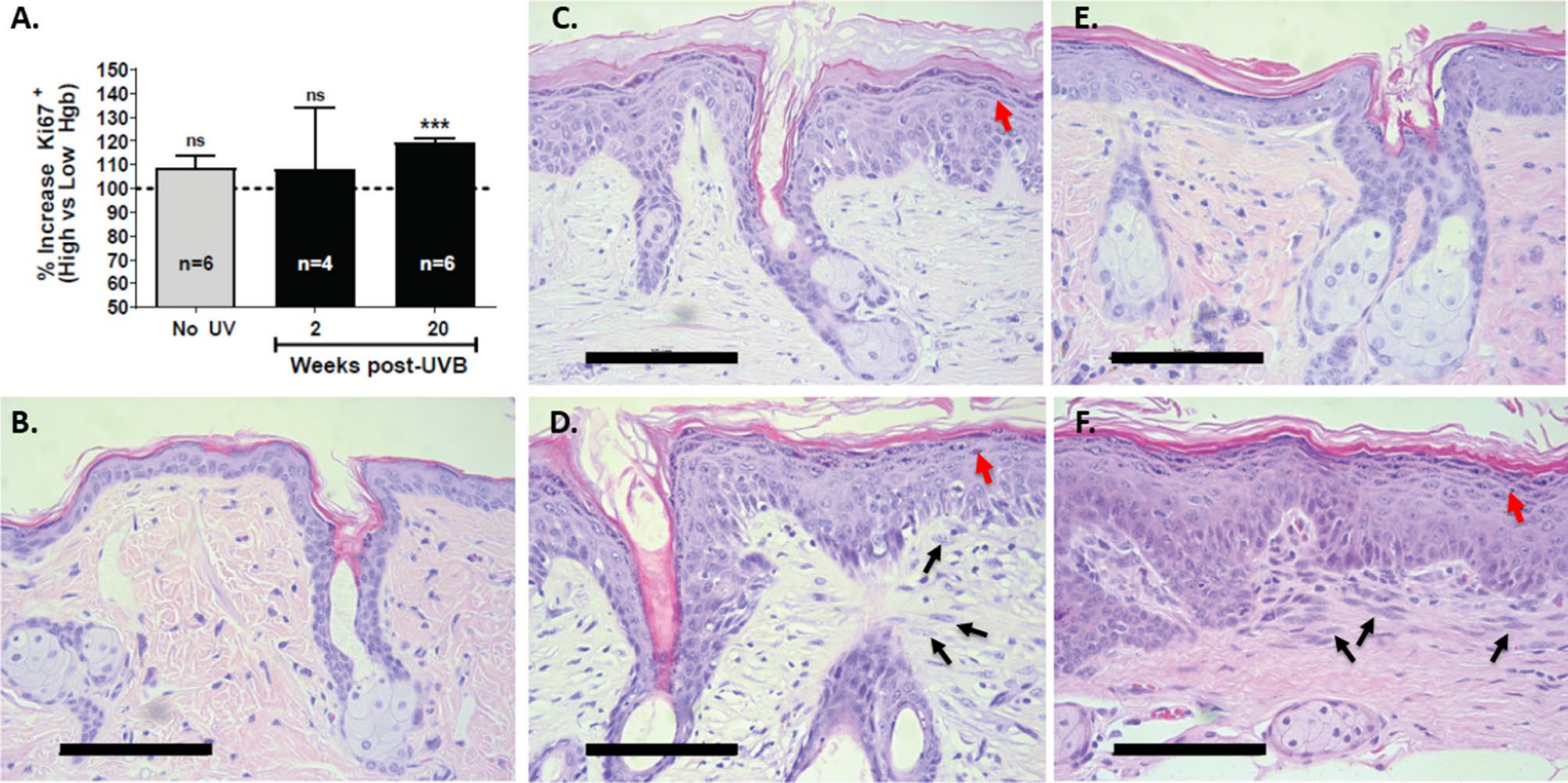
Epidermal hyperplasia persists only in areas of high hemoglobin (Hgb) content. As in Fig. 1, mice were treated with UVB for 10 weeks. Following imaging, Hgb content maps were generated and areas of high and low Hgb content were excised at both 2 and 20 weeks after stopping UV treatments. (**A**) *An increase in Ki67 immunopositivity is seen only in persistent hyperemic areas at 20 weeks after stopping UV treatments.* For each mouse, Ki67^+^ epidermal cells were counted in areas of both high and low Hgb content. The mean % difference for epidermal Ki67^+^ cells for areas of high Hgb content after subtracting areas of low Hgb content from the same mouse are shown. ***, *p* < 0.001, one sample *t*-test. (**B**) Representative hematoxylin & eosin (H&E) stained section of normal mouse skin (no UV treatment controls). (**C**–**F**) *Epidermal hyperplasia persists only in hyperemic areas.* Representative H&E stained sections from UV-treated mouse skin at 2 weeks (**C**,**D**) and 20 weeks (**E**,**F**) after stopping UV treatments. (**C**,**E**) represent areas of low hemoglobin content whereas (**D**,**F**) represent areas of high hemoglobin content. The red arrows highlight areas in which a prominent stratum granulosum is observed. The black arrows point out unusual dermal cells with enlarged, cigar-shaped nuclei. Scale bar represents 100 µm.

In Fig. 2A, we excised hyperemic and non-hyperemic foci that had developed at both 2 and 20 weeks after stopping UV treatments. We then performed Ki67^+^ immunolabeling to determine whether epidermal proliferative capacity differed when the skin was obtained from the hyperemic relative to the non-hyperemic areas. At two weeks after stopping UV treatments, shortly after the UV-induced inflammation (sunburn) had receded, there was no significant difference in Ki67^+^ epidermal cell counts between areas of high Hgb content relative to low Hgb content. The absence of a significant difference between hyperemic and non-hyperemic areas at this early time point after stopping UV treatments is consistent with all areas of the UV treated field receiving similar UV dosing. However, at 20 weeks after stopping UV treatments, long after the residual mitogenic effects of this exogenous promoter would have receded, Ki67^+^ epidermal cells were increased on average by 19.3 ± 4.57% in areas of hyperemia relative to non-hyperemic areas from the same mouse.

Epidermal hyperplasia is an indication of a sustained mitogenic signal and is seen as an increase in epidermal thickness. In mouse epidermis, it can also be seen as a more prominent stratum granulosum^19^. Figure 2B–F show representative hematoxylin & eosin stained skin sections in non-UV treated control mouse skin as well as skin excised from hyperemic and non-hyperemic skin at both early (2 weeks) and late (20 weeks) after stopping UV treatments. In agreement with the Ki67 data, epidermal hyperplasia was prominent in both hyperemic and non-hyperemic areas of the skin when the skin was excised shortly after stopping UV treatments (Fig. 2C,D). Given that Ki67 labeling was increased only in hyperemic areas relative to non-hyperemic areas when examined long after stopping UV treatments, we next examined whether epidermal hyperplasia persists only in hyperemic areas of skin isolated 20 weeks after stopping UV treatments. At this late time point, histologically evident epidermal hyperplasia was seen to persist in areas of hyperemia (Fig. 2F), but was largely absent in non-hyperemic areas (Fig. 2E). This data indicates that epidermal hyperplasia can persist long after stopping UV treatments. Importantly, this persistent epidermal hyperplasia was not randomly distributed throughout the treated field, but rather was confined to areas of overlying persistent dermal hyperemia. In addition, as noted by the black arrows in Fig. 2D,F, an increased number of dermal cells with enlarged cigar-shaped heterochromatic nuclei were seen just underlying the epidermis in hyperemic, but not non-hyperemic areas.

### UV-induced dermal hyperemic foci are characterized by senescent dermal cells

**Figure 3:**
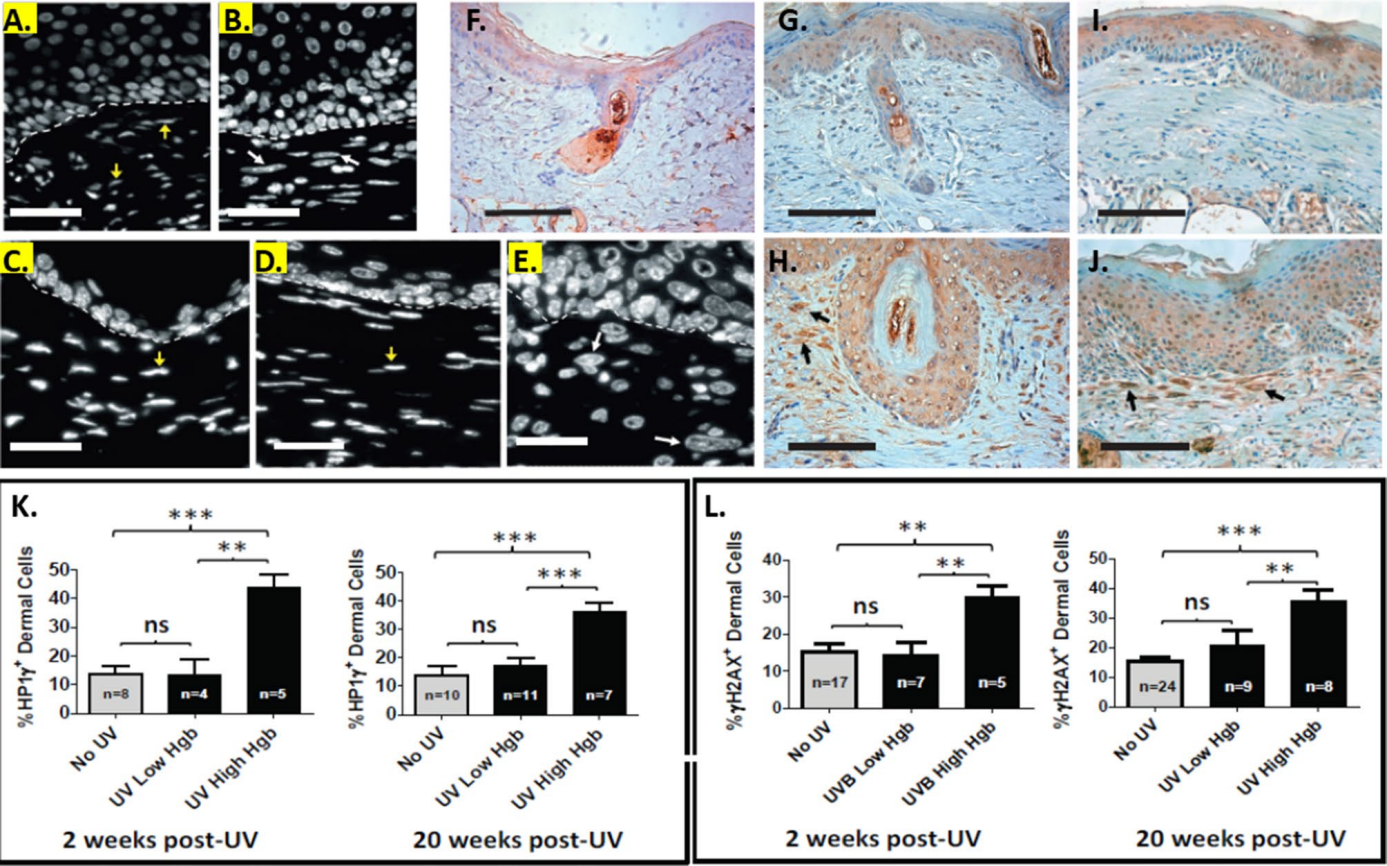
Persistent hyperemic foci exhibit evidence of dermal senescence. (**A**–**E**) *Dermal cells in areas of high Hgb content exhibit a heterochromatin pattern of DAPI staining.* After excision of skin from mice treated with and without UV at 2 and 20 weeks after stopping UV treatments, the sections were stained with DAPI to better demonstrate nuclear morphology. Nuclei of dermal cells showing a heterochromatin staining pattern are shown by white arrows while nuclei exhibiting a euchromatin pattern of DAPI staining are shown by yellow arrows. The hatched white line outlines the epidermal-dermal junction. The white scale bar represents 50 µm. (**A**) Skin from an area of low Hgb content 2 weeks after stopping UV treatments. (**B**) Skin from an area of high Hgb content 2 weeks after stopping UV treatments. (**C**) Control non-UV treated skin. (**D**) Skin from an area of low Hgb content 20 weeks after stopping UV. (**E**) Skin from an area of high Hgb content 20 weeks after stopping UV. **(F**–**J**) *Representative photomigrographs of skin following immunolabeling with anti-p16*^*INK4a*^* antibodies.* (**F**) Control non-UV treated epidermis. (**G**,**H**) Skin excised 2 weeks after stopping UV treatments from an area of low Hgb content (**G**) or from an area of high Hgb content (**H**). (**I**,**J**) Skin excised from a low Hgb area (**I**) or a high Hgb area (**J**) at 20 weeks after stopping UV treatments. Black arrows in (**H**,**J**) show dermal cells with enlarged nuclei labeling positive for p16^INK4a^. The black scale bars represent 100 µm. (**K**) *HP1γ*^+^ *dermal cells are increased in hyperemic areas at both 2 and 20 weeks after stopping UV treatments.* Immunofluorescent (IF) labeling of formalin-fixed skin sections was performed using anti-HP1γ and anti-pancytokeratin (CK) antibody. The data depicts the % of dermal cells positive for HP1γ nuclear labeling. (**L**) *Dermal cells positive for nuclear γH2AX are also increased in hyperemic areas only at 2 and 20 weeks post-UV.* IF was performed for both nuclear γH2AX immunolabeling and CK. The data shown is the percentage of γH2AX^+^ dermal cells relative to all CK negative cells. (ns = non-significant; ** = *p* < 0.01; *** = *p* < 0.001).

Enlarged heterochromatic nuclei are characteristic of senescent cells^16^. We therefore examined stromal nuclear morphology following 4′,6-diamidino-2-phenylindole (DAPI) staining (Fig. 3A–E). Two and 20 weeks after stopping UV treatments, we observed that many dermal cells within hyperemic areas exhibited enlarged nuclei with a heterochromatin pattern of DAPI fluorescence (Fig. 3C,E). In non-UV treated mouse skin (Fig. 3A) and in low Hgb areas at both 2 weeks (Fig. 3B) and 20 weeks (Fig. 3D) after stopping UV treatments, dermal cell nuclei largely had a euchromatin pattern of DAPI-uptake.

Nuclear accumulation of the cyclin dependent kinase inhibitor p16^Ink4a^ is a characteristic of post-mitotic senescent cells^16^. In Fig. 3F–J, we show representative photomicrographs of p16^Ink4a^ immunolabeling. Dermal cells labeling positive for p16^Ink4a^ were infrequent in non-UV treated epidermis (Fig. 3F), or in non-hyperemic areas of skin at either 2 or 20 weeks after stopping UV treatments (Fig. 3G,I, respectively). In contrast, dermal cells with enlarged p16^Ink4a^ positive nuclei were prominently observed in hyperemic areas from skin obtained at both 2 (Fig. 3H) and 20 (Fig. 3J) weeks after the cessation of UV.

We next examined nuclear expression of heterochromatin protein 1 homolog gamma (HP1γ), also known as chromobox protein homolog 3 (*Cbx3*). Increased punctate nuclear labeling of HP1γ is a marker of senescence-associated heterochromatic foci (SAHF)^16^. As seen in Fig. 3K and Supplemental Figs. S1 & S2, HP1γ^+^ dermal cells were significantly elevated in areas of high Hgb content relative to areas of low Hgb content at both 2 and 20 weeks after stopping UV treatments. In contrast, there was no significant difference in HP1γ^+^ epidermal cells in the UV-treated areas at high or low Hgb content at either time point (Supplemental Figs. S1 & S2).

Another characteristic feature of senescence is a persistent DNA damage response (DDR)^16^. The DDR is heralded by the appearance of punctate nuclear foci that stain positive for the histone protein H2AX phosphorylated at serine 139 (termed γH2AX)^16,20^. In Fig. 3L and Supplemental Figures S3 & S4, we show that dermal cells exhibiting punctate nuclear staining for γH2AX are significantly enriched only in areas of high Hgb content. This was observed at both 2 and 20 weeks after stopping UV treatments. As with HP1γ immunolabeling, there was no significant difference in γH2AX^+^ epidermal cells in the UV-treated areas at high or low Hgb content at either time point (Supplemental Figs S3 & S4).

### UV-induced persistent dermal hyperemic foci correlate with the persistence of p53^+^ epidermal patches

**Figure 4:**
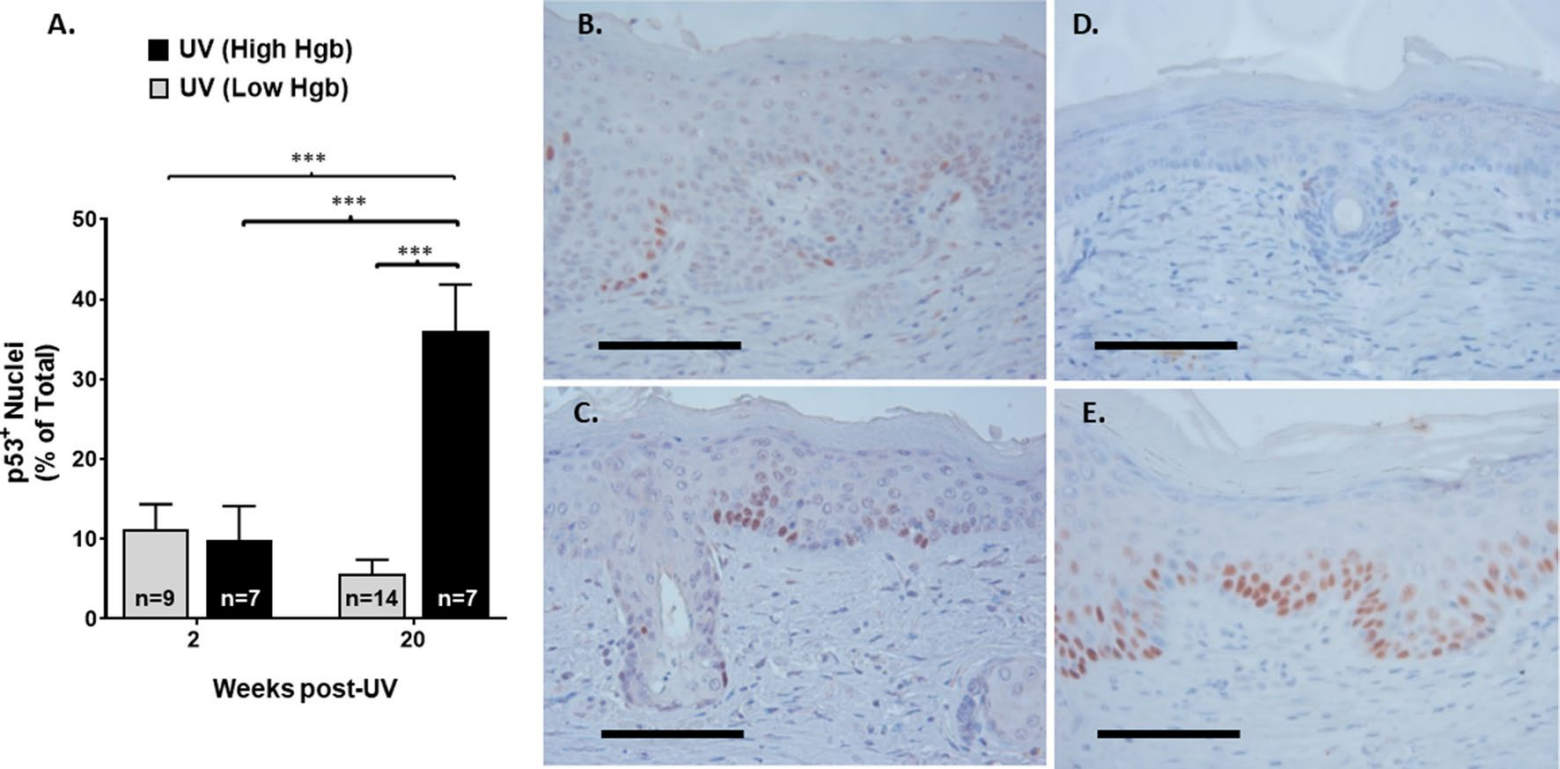
Epidermal p53^+^ cells are elevated equivalently in both hyperemic and non-hyperemic areas 2 weeks after stopping UV treatments but are increased only in hyperemic areas when examined 18 weeks later. Skin excised from areas of high and low Hgb content were used to quantitate p53^+^ epidermal cells by immunohistochemistry at both 2 and 20 weeks after stopping UV treatments. Non-UV treated control skin had negligible numbers of p53^+^ epidermal cells at either time point (not shown). (**A**) No significant difference was observed between hyperemic and non-hyperemic foci for p53^+^ epidermal cell numbers at 2 weeks post-UV cessation. At 20 weeks post-UV, there was a significant enrichment of p53^+^ cells in the hyperemic foci. (*** = *p* < 0.001). (**B**–**E**) Representative photomicrographs of p53^+^ nuclear labeling in hyperemic areas (**C**,**E**) and non-hyperemic areas (**B**,**D**) at both 2 weeks (**B**,**C**) and 20 weeks (**D**,**E**) after stopping UV. The black scale bars represent 100 µm.

We next examined epidermal p53 immunopositivity within the epidermis overlying hyperemic and non-hyperemic areas at both 2 and 20 weeks after stopping UV treatments. In Fig. 4A and depicted in the representative photomicrographs of nuclear p53 immunolabeling seen in Fig. 4B & C, we show that there is no difference in the percentage of epidermal p53^+^ cells between hyperemic and non-hyperemic areas when examined only two weeks after stopping UVB treatments. As with the Ki67 data in Fig. 2A, this data indicates that the cumulative UV dosing was equivalent throughout the treated field. In contrast, at 20 weeks following the discontinuation of UV treatments, p53^+^ epidermal cells not only persisted but were significantly enriched only in areas overlying the persistent dermal hyperemic field (Fig. 4A,D,E). Only rare p53^+^ cells were seen in non-UV treated controls (not shown). Negligible p53 immunolabeling in non-UV treated skin is consistent with previous observations^21^.

## Discussion

**Figure 5:**
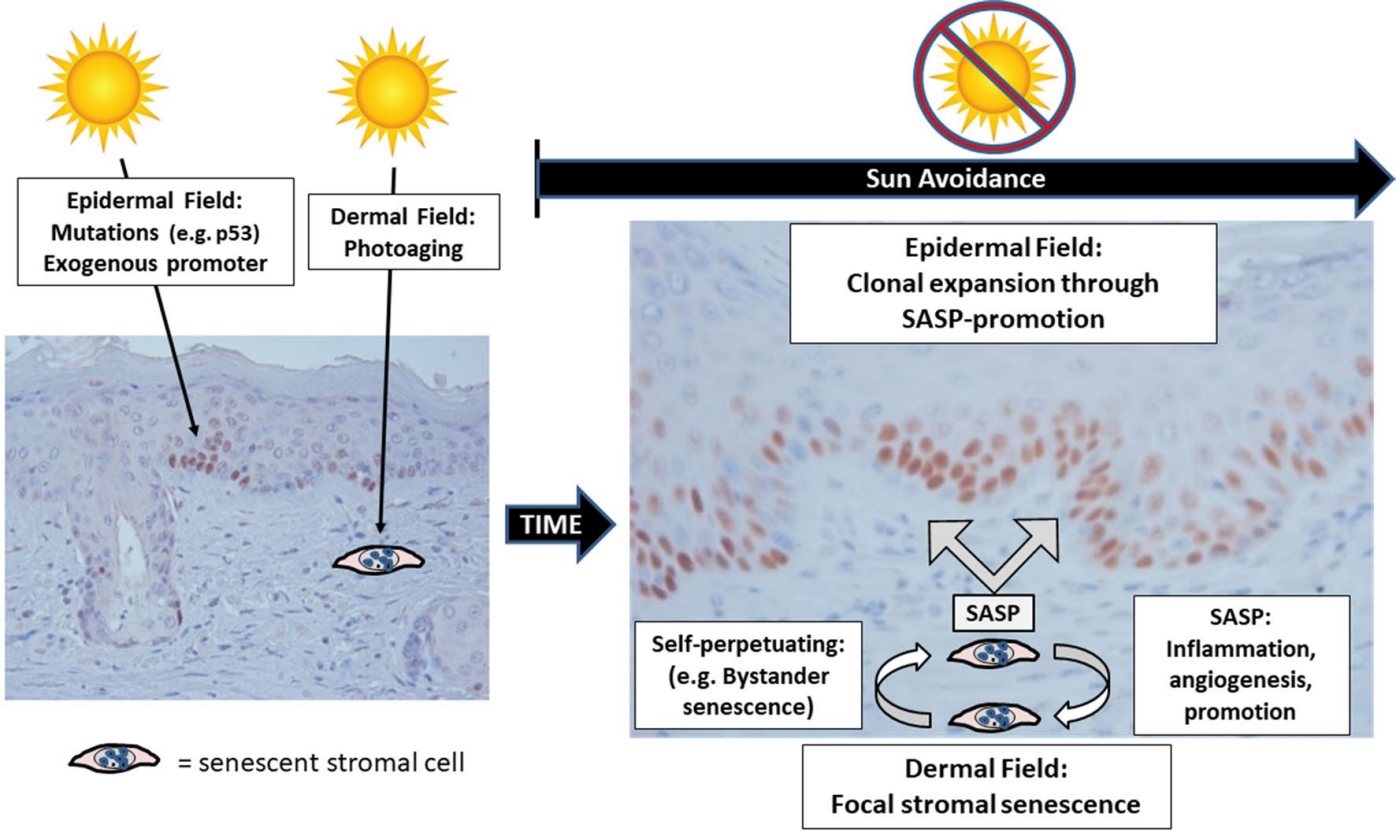
Hypothetical Two Field Model for sustained cancer risk in the absence of continued UV treatment. Continuous UV exposures elicits mutations within the overlying epidermis. UV also acts as an exogenous promoting agent that stimulates clonal expansion of the overlying mutated epidermal cells. In the dermis, UV promotes photoaging and inflammation. Once UV exposures cease, the tumor promoting actions of UV exposures are extinguished. However, focal areas of dermal senescence develop in concert with dermal hyperemia. Once established, these dermal senescent hyperemic foci persist and expand. As part of the so-called senescence-associated secretory phenotype (SASP), senescent cells are known to secrete multiple cytokines, chemokines, mitogens, and angiogenic factors. The SASP could account for both the sustained inflammatory angiogenesis that is characteristic of the dermal cancer field, as well as to sustain a promoting environment for the clonal expansion of epidermal cells containing key driver mutations, such as p53. Persistence and expansion of the senescent hyperemic dermal foci could occur through bystander senescence.

A key feature of our study is the ability to map the cancer field early in the premalignant period prior to tumor formation. Using this powerful approach, we provide data using multiple senescence markers that dermal senescence is a characteristic feature of hyperemic foci. This senescent dermal phenotype is observed early and persists. We also show that while epidermal hyperplasia and p53^+^ cells are initially randomly distributed throughout the UV-treated field, their persistence after 20 weeks is not random, but rather is restricted to sites of dermal hyperemia (dermal cancer field). Since hyperemic foci represent approximately 20% of the UV-treated imaging area^9^, this suggests that clonal expansion of p53^+^ clones occurs only within a relatively small proportion of the epidermis. Thus, these observations challenge current stochastic models of epithelial cancer field persistence that have relied solely on a random examination of the epidermal mutation field. This data also suggests that once established, continued UV treatments are no longer necessary to maintain the cancer field. We propose that the persistent senescent dermal field, once established, is sufficient to provide the tumor promoting functions that would otherwise require continued UV exposures (Fig. 5). This model also suggests that a greater understanding of the dermal field might result in new targets for cancer chemoprevention, particularly in those who have altered their lifestyle to limit continued UV exposures.

While the idea that senescent stromal cells can contribute to tumor growth is not new, our studies add to current knowledge by introducing the idea that a senescent stromal field could contribute to early epithelial field persistence in the absence of continued promoter exposures and is therefore critical to persistent cancer risk.

The idea that a senescent dermal field could drive epithelial field persistence is suggested by previous observations. First, senescent stromal cells are often seen in human and mouse tumors^15^. Second, co-transfer studies indicate that senescent fibroblasts can promote the growth, invasiveness and metastasis of tumor cells through production of the so called senescence associated secretory phenotype (SASP)^14^. Third, selective killing of p16^Ink4a^ expressing cells was shown to reduce tumorigenesis in mice^22^. Finally, studies by G. Paolo Dotto and co-workers showed that selective loss of Notch signaling by knockout of RBP-Jκ in mesenchymal cells of mice can elicit dermal inflammation, spontaneous skin tumors and multi-focal areas of hyperplastic epidermis^10,11^. They also observed that loss of RBP-Jκ signaling led to a senescent dermal phenotype, that UVA treatment suppressed RBP-Jκ signaling, and that senescent stromal cells could be found in human pre-malignant skin lesions and actinic keratoses, but were infrequent in invasive SCCs^11^.

While the studies by Dotto and co-workers are intriguing, they failed to demonstrate whether senescence is a critical determinant of the pro-tumorigenic activity or simply a secondary effect of loss of RBP-Jκ signaling. While the investigators showed that UVA could suppress RBP-Jκ signaling in fibroblasts, the investigators failed to show whether the loss of RBP-Jκ signaling in UVA treated fibroblasts persists after stopping UVA. Thus, it is unclear how their findings would translate to persistent cancer risk following the cessation of UV treatments. Moreover, while loss of RBP-Jκ signaling was demonstrated uniformly throughout the dermis, both inflammatory infiltrates and epidermal hyperplasia (assessed by 5-ALA uptake) were not uniform, but rather multi-focal^17^. Since the investigators failed to determine whether the dermis underlying the areas of increased 5-ALA uptake also exhibited a senescent phenotype, it is unclear what role senescence played in early field formation. In addition, the investigators also demonstrated that tumor-dependent signaling was necessary to drive the generation of CAFs through loss of p53 signaling within the fibroblasts^11^. Mitotically active CAFs have secretory activity that exhibits considerable overlap with the SASP^23^. It is therefore unclear whether tumor-modulating activity is driven by the senescent phenotype or whether this activity is dependent on tumor-dependent CAF formation.

A senescent dermal environment may also explain our observation that the hyperemic dermal field not only persists, but also expands over time in the absence of any further UV treatments^9^. Senescent cells have been shown to promote senescence in non-senescent cells through a so-called bystander effect^24^. Bystander cells themselves, upon undergoing senescence, also produce senescence-inducing SASP mediators^24^. This introduces a potential feed-forward mechanism that could drive both the persistence and expansion of the dermal cancer field.

A limitation of our study is that we have not established that the senescent dermis plays a causal role in the observed persistent hyperplasia and the expansion of p53^+^ epidermal cells. To demonstrate causality, future studies are needed to selectively eradicate senescent stromal cells within the dermal field and show that this suppresses dermal hyperemia, overlying epidermal clonal expansion, and subsequent tumor formation. Unfortunately, while it is possible to induce senescent cell death non-selectively by either genetic approaches (e.g. p16-3MR mice^25^, INK-ATTAC mice^26^) or senolytic agent treatment, there are no current methods that can reliably deplete senescent cells within a particular tissue or cell type. This is important, as tumors may arise from senescent premalignant cells through the loss of cell cycle checkpoints^27,28^. In addition, loss of mitotically-inactive senescent epidermal cells can trigger adjacent mitotically competent mutated stem cells to undergo proliferative clonal expansion by loss of cell-contact growth inhibition (e.g. frontier hypothesis)^29^. Thus, an early depletion of senescent epidermal cells could directly affect the epithelial field independent of the senescent stromal compartment.

A second limitation of our study is that all of our work has been completed in mice. Given that mice age differently than humans and their response to UV exposures may also differ, these studies may not accurately reflect the human condition. Of relevance to this manuscript, potential species differences can be noted in senescence induction. While a retinoblastoma (*Rb1*) and p16^Ink4a^ pathway is necessary for senescence induction in mouse fibroblasts, the human *RB1* paralog *RB2* is more important in humans, acting through p27 and p16^INK4A^ to induce senescence in human fibroblasts^30^. Future work is necessary in human subjects to broaden the potential impact of our studies.

In conclusion, while there has been a great deal of work on examining the cancer field in mouse models of chemical and UV-induced carcinogenesis, most of this work has been done examining how continuous applications of an exogenous promoting agent (either a chemical tumor promoter or UV) drive the formation of the cancer field. This scenario would be common to individuals with job or recreational-related persistent sun exposure. In this setting, the promoting aspect of the senescent dermal field would likely have limited impact. The innovation of our approach is that we examine an alternative scenario that is very common in humans. Individuals with significant photodamage frequently reduce their sun exposure due to the desire to reduce the cosmetic damage secondary to photoaging or to reduce their risk for skin cancer formation. In the absence of the continued exposure to the promoting aspects of the sun, our study provides a novel testable model for how the cancer field (and cancer risk) persists.

## Methods

### Animal studies

Out-bred euthymic and immunocompetent SKH-1 (SKH1-Hrhr) hairless albino mice were purchased from Charles Rivers (Wilmington, MA). A total of 24 female mice at 7–10 weeks of age at the beginning of the experiment were used. Male mice were not used since fighting behavior results in increased skin wounds that would interfere with our imaging studies. Mice were housed under specific pathogen-free conditions at the Indiana University School of Medicine. Animals were housed in a constant temperature room at a maximum of 5 mice per cage in standard microisolator cages, had a 12 h light/dark cycle and access to food and water ad libitum. Animal use protocols were approved by the Indiana University School of Medicine Institutional Animal Care and Use Committee (IACUC). All animal studies follow guidelines established by the National Institutes of Health.

### Chronic UV treatments and intravital imaging

Mice were treated for 10 weeks with UVB (2240 J/m^2^) three times per week as previously described^9^. After 10 weeks, UVB treatments were stopped, and imaging for hemoglobin content was performed at 2 and 20 weeks thereafter as described^9^.

### Immunolabeling

Immunofluorescent staining of formalin-fixed paraffin-embedded mouse skin following heat-induced antigen retrieval was performed using the following antibodies: HP1γ (Cbx3) immunolabeling was done using rabbit anti-Cbx3 (1:250, Cat#IHC-00204, Bethyl Laboratories). γH2AX immunolabeling was done using monoclonal rabbit anti-γH2AX (1:100, Bethyl Laboratories, Cat#A700-053). Ki67 immunolabeling was done with rabbit monoclonal anti-Ki67 (1:200, clone SP6, ThermoScientific). For p53, rabbit polyclonal anti-p53 (1:1000, NCL-p53-cmSP, Leica Biosystems) was utilized. For p16^Ink4a^, rabbit polyclonal anti-p16 (1:100, M-156, Santa Cruz Biotechnology) was utilized. Immunofluorescence labeling for pan-cytokeratin expression was performed as previously detailed^31^. Chromogenic immunolabeling using DAB as a substrate was done for p53 immunolabeling. Since p53^+^ cells frequently persist as patches of p53^+^ cells, we selected the five 400 × fields per section that had the greatest number of p53^+^ cells. Epidermal and dermal Ki67, HP1γ and γH2AX was assessed from 4–5 random 200 × fields per section after co-labeling epidermal cells using pan-cytokeratin antibodies using TissueQuest image cytometery software (Version 3.0.1120.013720110307.01, TissueGnostics, Vienna, Austria).

### Statistical analysis

Statistical analyses were performed by using GraphPad Prism 5 (La Jolla, CA, USA). Statistical significance was assigned at a *P* value less than 0.05. For multiple comparison testing, statistical analysis was performed using 1-way ANOVA with Tukey’s multiple comparison test. For Fig. 2A,B, a one sample t-test was used to assess a statistical difference from 100%.

## Supplementary information

Supplementary Information
