# Supplementary material for: Evidence for a non-stochastic two-field hypothesis for persistent skin cancer risk
Source: Sci Rep. 2020 Nov 5;10:19200. doi: 10.1038/s41598-020-75864-2 (PMC7645611; doi:10.1038/s41598-020-75864-2)

Supplemental information:

Evidence for a non-stochastic two-field hypothesis for persistent cancer risk

Raymond L Konger, Lu Ren, Ravi P. Sahu, Ethel Derr-Yellin, and Young L. Kim

Supplemental Figure S1: HP1γ immunofluorescence at 2 weeks after stopping UV treatments.

Supplemental Figure S2: HP1γ immunofluorescence at 20 weeks after stopping UV treatments.

Supplemental Figure S3: γH2AX immunofluorescence at 2 weeks after stopping UV treatments.

Supplemental Figure S4: γH2AX immunofluorescence at 20 weeks after stopping UV treatments.

Supplemental Figure S1: HP1γ immunofluorescence at 2 weeks after stopping UV treatments. Representative photomicrographs are shown for HP1γ staining in the dermis (pan-cytokeratin negative) and the epidermis (pan-cytokeratin positive) of non-hyperemic (A) and hyperemic (B) foci. C). The percentage of pan-cytokeratin^{+} cells that were also HP1γ^{+} are shown. There was no significant difference in HP1γ^{+} cells with the epidermal field in non-UV treated controls (No UV) relative to UV-treated hyperemic (UV High Hgb) and non-hyperemic (UV Low Hgb) foci. Data was obtained from n=8, 4, and 5 different tissue sections for the No UV, UV Low Hgb, and UV High Hgb areas, respectively. Differences were non-significant by One-way analysis of variance with Tukey's Multiple Comparison Test. D.) Enlarged inset from panel B showing punctate nuclear HP1γ immunolabeling in enlarged cigar-shaped dermal nuclei (arrows). Scale bar (A&B) = 100 µm; (D) = 50 µm.

Red = HP1γ
Green = pan-cytokeratin
Blue = DAPI

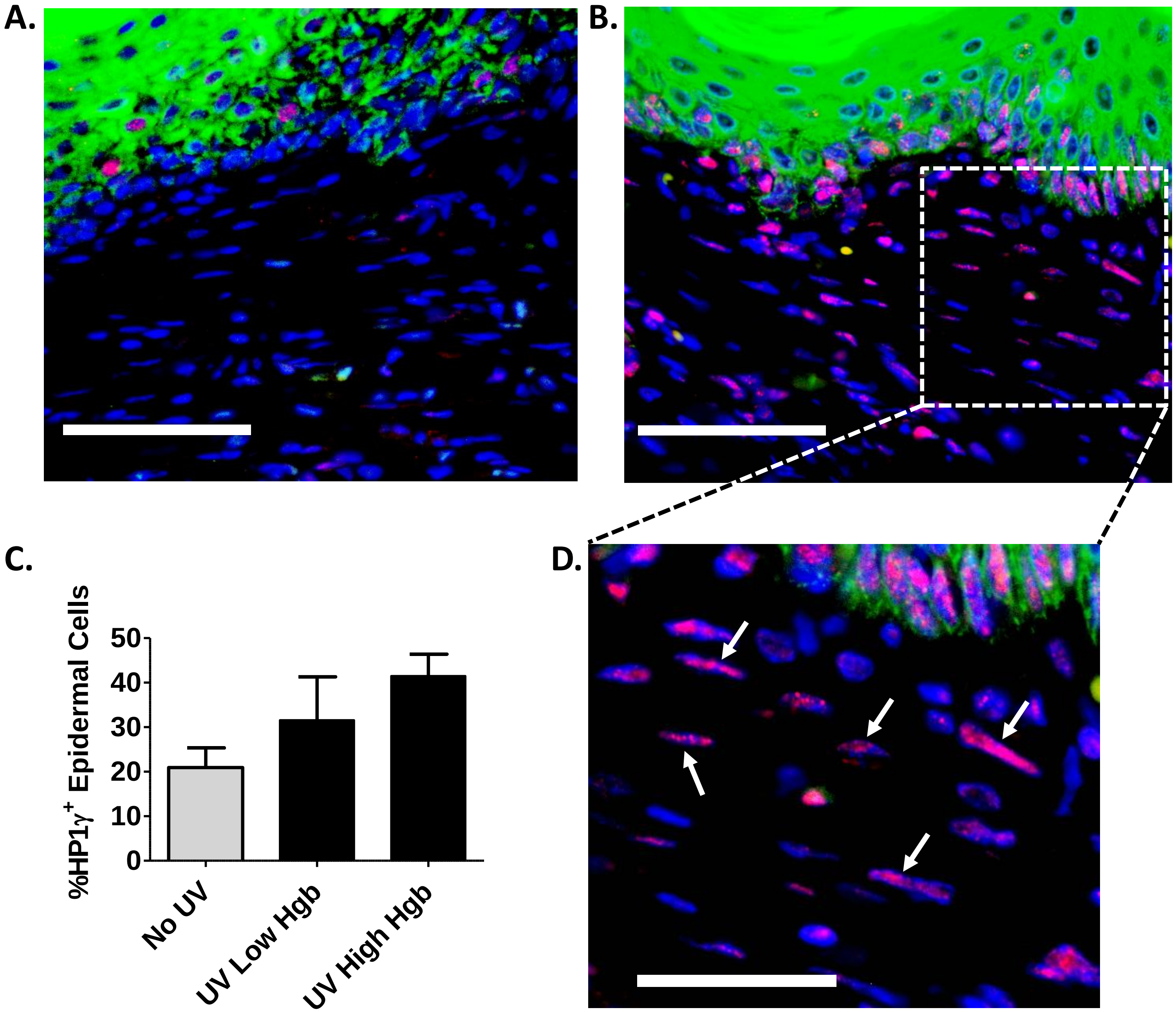

Supplemental Figure S2: HP1γ immunofluorescence at 20 weeks after stopping UV treatments. Representative photomicrographs are shown for HP1γ staining in the dermis (pan-cytokeratin negative) and the epidermis (pan-cytokeratin positive) of non-hyperemic (A) and hyperemic (B) foci. C). The percentage of pan-cytokeratin⁺ cells that were also HP1γ⁺ are shown. There was no significant difference in HP1γ⁺ cells with the epidermal field in non-UV treated controls (No UV) relative to UV-treated hyperemic (UV High Hgb) and non-hyperemic (UV Low Hgb) foci. Data was obtained from n=10, 11, and 7 different tissue sections for the No UV, UV Low Hgb, and UV High Hgb areas, respectively. Differences were non-significant by One-way analysis of variance with Tukeys's Multiple Comparison Test. D.) Enlarged inset from panel B showing punctate nuclear HP1γ immunolabeling in enlarged cigar-shaped dermal nuclei (arrows). Scale bar (A&B) = 100 µm; (D) = 50 µm.

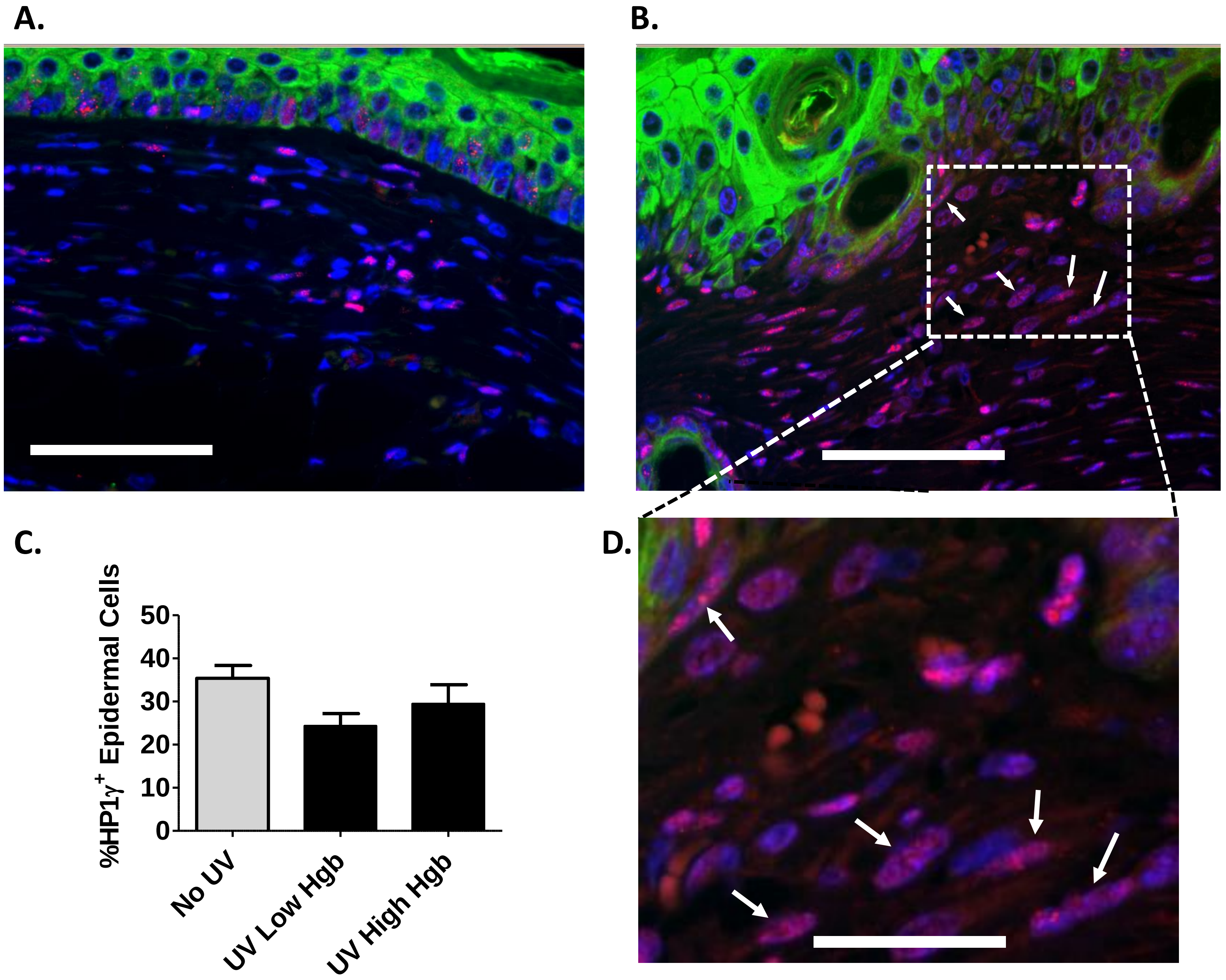

Red = HP1γ
Green = pan-cytokeratin
Blue = DAPI

Supplemental Figure S3: γH2AX immunofluorescence at 2 weeks after stopping UV treatments. Representative photomicrographs are shown for γH2AX staining in the dermis (pan-cytokeratin negative) and the epidermis (pan-cytokeratin positive) of non-hyperemic (A) and hyperemic (B) foci. C). The percentage of pan-cytokeratin$^+$ cells that were also γH2AX $^+$ are shown. There was no significant difference in γH2AX $^+$ cells with the epidermal field in non-UV treated controls (No UV) relative to UV-treated hyperemic (UV High Hgb) and non-hyperemic (UV Low Hgb) foci. Data was obtained from n=24, 8, and 9 different tissue sections for the No UV, UV Low Hgb, and UV High Hgb areas, respectively. Differences were non-significant by One-way analysis of variance with Tukey's Multiple Comparison Test. D.) Enlarged inset from panel B showing punctate nuclear γH2AX immunolabeling in enlarged cigar-shaped dermal cell nuclei (arrows). Scale bar (A&B) = 100 µm; (D) = 50 µm.

Red = γH2AX
Green = pan-cytokeratin
Blue = DAPI

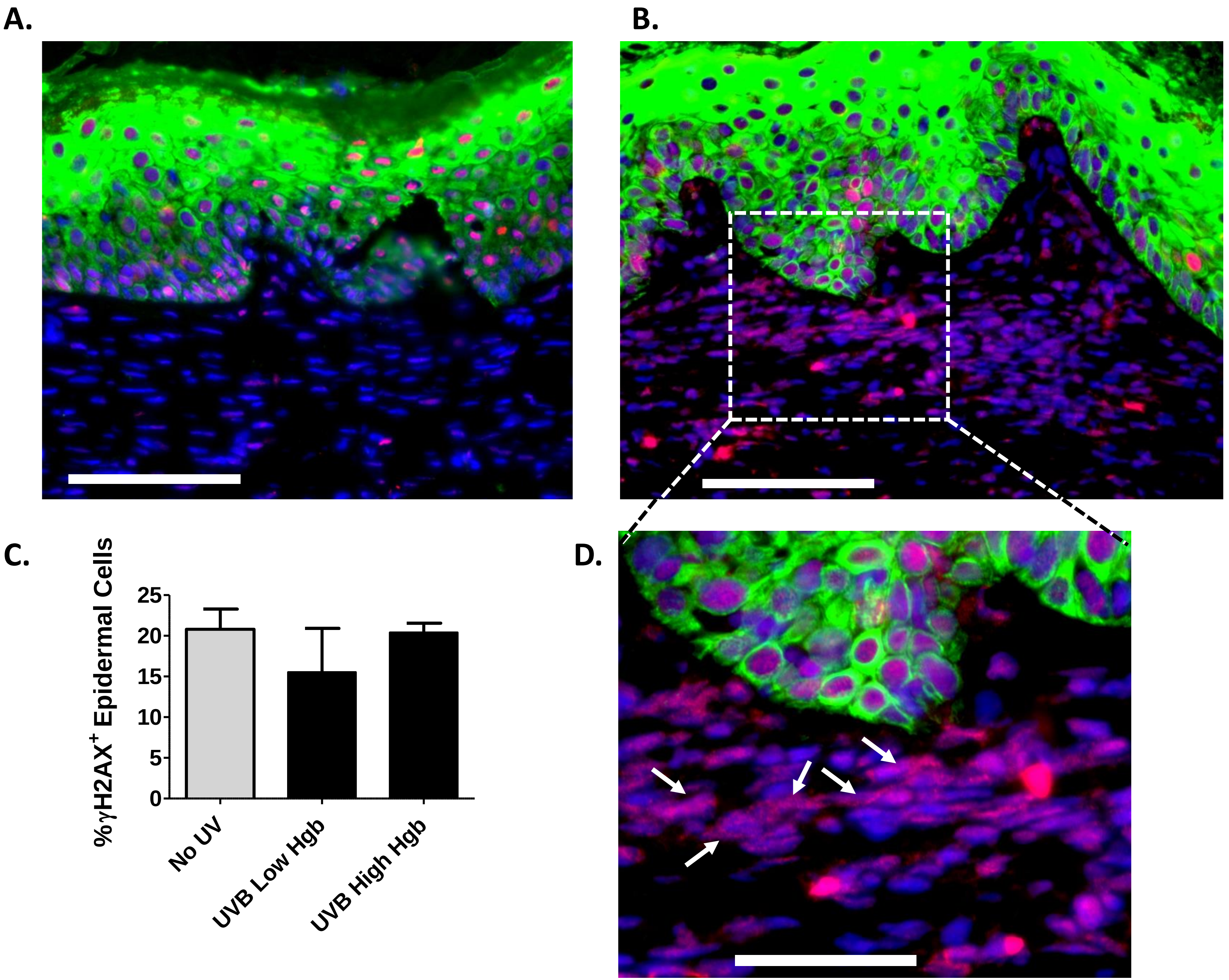

Supplemental Figure S4: γH2AX immunofluorescence at 20 weeks after stopping UV treatments. Representative photomicrographs are shown for γH2AX staining in the dermis (pan-cytokeratin negative) and the epidermis (pan-cytokeratin positive) of non-hyperemic (A) and hyperemic (B) foci. C). The percentage of pan-cytokeratin$^+$ cells that were also γH2AX$^+$ are shown. There was no significant difference in γH2AX$^+$ cells with the epidermal field in non-UV treated controls (No UV) relative to UV-treated hyperemic (UV High Hgb) and non-hyperemic (UV Low Hgb) foci. Data was obtained from n=24, 8, and 9 different tissue sections for the No UV, UV Low Hgb, and UV High Hgb areas, respectively. Differences were non-significant by One-way analysis of variance with Tukey's Multiple Comparison Test. D.) Enlarged inset from panel B showing punctate nuclear γH2AX immunolabeling in enlarged cigar-shaped dermal cell nuclei (arrows). Scale bar = 100 µm; (D) = 50 µm.

Red = γH2AX
Green = pan-cytokeratin
Blue = DAPI

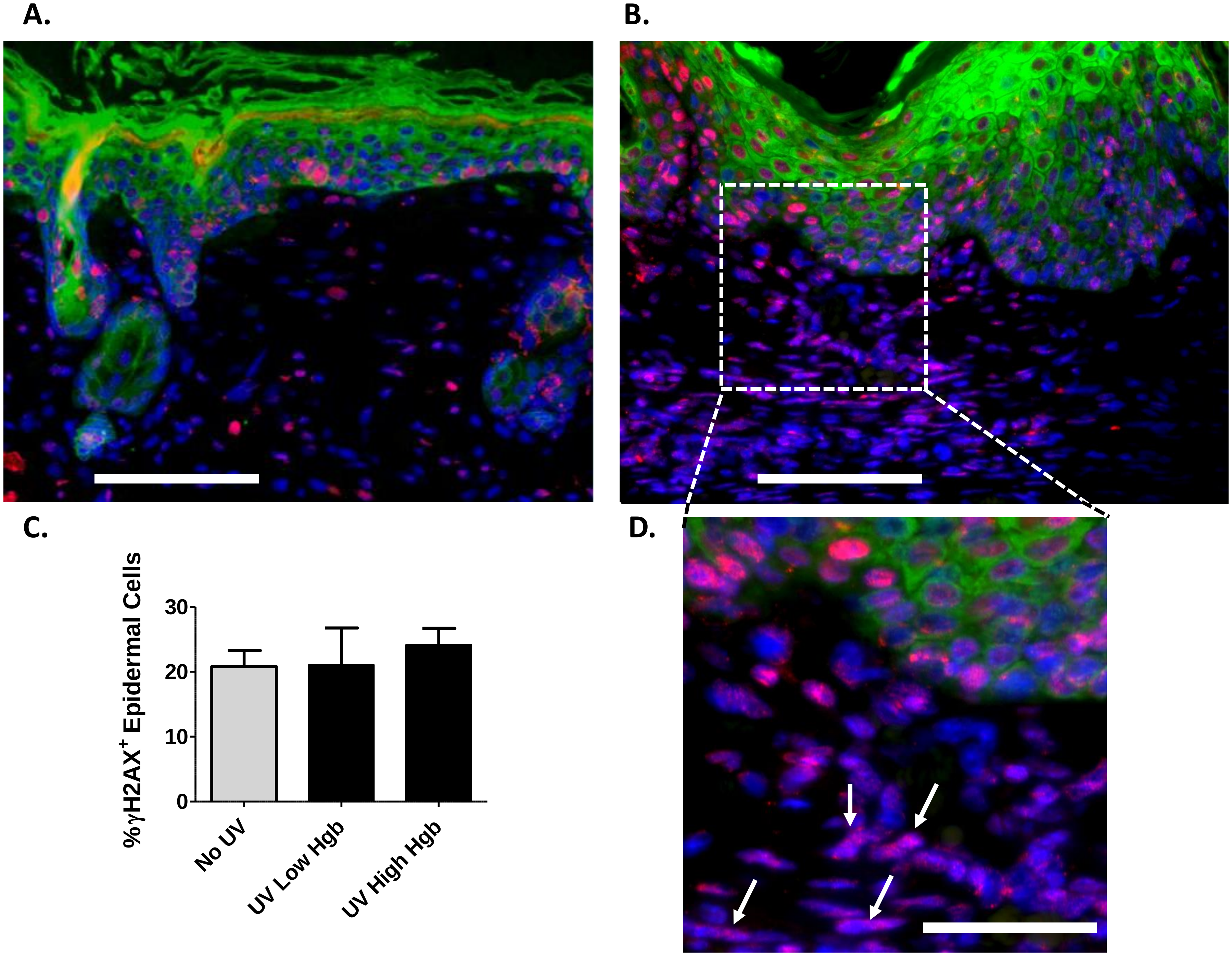

Supplement: Supplementary file 1 — Supplementary Information [file 41598_2020_75864_MOESM1_ESM.pdf]
